# Experimental warming increases ecosystem respiration by increasing above-ground respiration in alpine meadows of Western Himalaya

**DOI:** 10.1038/s41598-021-82065-y

**Published:** 2021-01-29

**Authors:** Pankaj Tiwari, Pamela Bhattacharya, Gopal Singh Rawat, Ishwari Datt Rai, Gautam Talukdar

**Affiliations:** 1 Wildlife Institute of India, Dehradun, Uttarakhand 248001 India; 2 Indian Institute of Remote Sensing, ISRO, Dehradun, Uttarakhand 248001 India

**Keywords:** Climate-change impacts, Climate-change ecology, Atmospheric science

## Abstract

Alpine ecosystems in the Himalaya, despite low primary productivity, store considerable amount of organic carbon. However, these ecosystems are highly vulnerable to climate warming which may stimulate ecosystem carbon efflux leading to carbon-loss and positive feedback. We used open-top chambers to understand warming responses of ecosystem respiration (ER) and soil respiration (SR) in two types of alpine meadows viz., herbaceous meadow (HM) and sedge meadow (SM), in the Western Himalaya. Experimental warming increased ER by 33% and 28% at HM and SM, respectively. No significant effect on SR was observed under warming, suggesting that the increase in ER was primarily due to an increase in above-ground respiration. This was supported by the warming-induced increase in above-ground biomass and decrease in SR/ER ratio. Soil temperature was the dominant controlling factor of respiration rates and temperature sensitivity of both ER and SR increased under warming, indicating an increase in contribution from plant respiration. The findings of the study suggest that climate warming by 1.5–2 °C would promote ER via increase in above-ground respiration during the growing season. Moreover, net C uptake in the alpine meadows may increase due to enhanced plant growth and relatively resistant SR under warming.

## Introduction

Fifth assessment report of IPCC predicts an increase of 0.3–4.8 °C global mean temperature by the year 2100^1^. The increase in temperature will be more prominent at higher altitudes, specifically alpine regions^2–5^ which store a large quantity of soil organic carbon (SOC) due to low temperature associated slow decomposition and low turnover rates^6–8^. Stored SOC is released as atmospheric CO_2_ by soil respiration (SR). SR accounts for both autotrophic respiration by root and root-associated microbes and heterotrophic respiration by free-living soil microbes that decompose plant litter and stored SOC. Globally, ecosystem respiration (ER) is the largest land to air CO_2_ flux and accounts for about ~ 90 PtG C per year, which is approximately nine times the anthropogenic CO_2_ emission^9,10^. ER (contributed by above ground plant respiration and SR) increases with increasing temperature under adequate moisture conditions^11,12^. However, ER is strongly influenced by quality and quantity of SOC, vegetation type, standing biomass and soil type^13–16^.

Variations in atmospheric CO_2_ are an effect of climate-induced changes in ER^17–19^ and a small shift in the flux can have a profound impact on the global carbon balance^19–22^. Understanding the response of ER and its components to warming is important in evaluating the global carbon cycle and predicting future changes in atmospheric CO_2_ levels^23–25^. Studies on impacts of warming on ER and SR have come up with contrasting results showing positive^25,26^, negative^27,28^ and even neutral responses^12^. These variations have originated mainly due to inherent differences in ecosystem properties and heterogeneity in soil biota^29–31^. For this reason, warming responses of ER remains one of the major sources of uncertainty in Earth System Models for climate projections^32–34^. Quantitative studies are required under varying habitat conditions to assess impacts of ER and its components to climate and vice versa.

Alpine region occupies nearly 33% of the geographical area in the Himalaya and store a considerable amount of SOC^35,36^. A large proportion of the alpine region in Himalaya are covered by meadows that are broadly classified as alpine moist herbaceous meadows, and alpine dry meadows dominated by graminoids i.e., grasses and sedges^36,37^. These habitats are of much ecological interest owing to predominance of specialized plant-forms adapted under harsh climatic conditions, their ability to translocate much of the synthesized carbon to underground parts as a survival strategy under prolonged and severe winters and their sensitivity to changing climate^36–38^. ER in these habitats is expected to increase with rising temperature setting positive feedback to climate warming^12,39^. The alpine habitats in particular and Himalayan region as a whole is said to have experienced an increase of 0.9 °C average temperature during 1901–2003 and continues to warm^40–42^. However, studies pertaining to assess impacts of warming on ecosystem functioning and patterns of C emission, especially from alpine region of Himalaya are lacking^43^. This is the first study that attempts to investigate the effects of climate warming on ER and SR in the alpine region of Himalaya based on warming experiment. We measured ER and SR in response to experimental warming in two types of alpine meadows in Western Himalaya viz., herbaceous meadow (HM) and sedge meadow (SM) during the growing season of 2019. We hypothesized that (1) warming would stimulate both ER and SR, (2) warming-induced decrease in soil moisture would limit increase in ER and SR, and (3) temperature sensitivity of both ER and SR would decrease under warming in both types of alpine meadows.

## Results

### Effect of warming on microclimate

**Figure 1:**
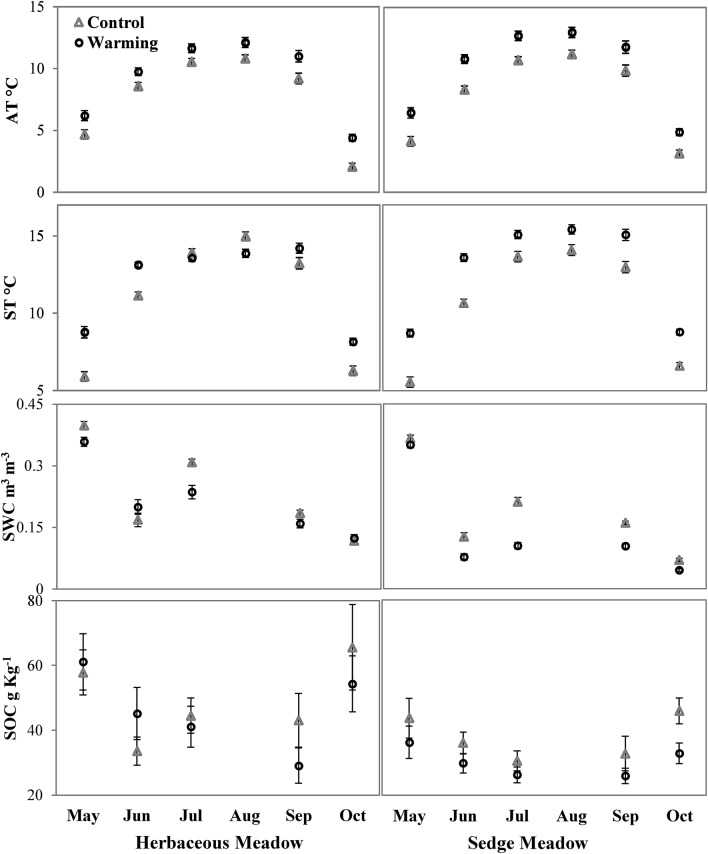
Warming effect on air temperature (AT), soil temperature (ST), volumetric soil water content (SWC) and soil organic carbon (SOC) at Herbaceous and Sedge Meadows. Data show mean ± S.E.

Experimental warming significantly increased air temperature (AT) at 30 cm height by 1.49 °C ± 0.37 °C (p < 0.001) and 1.9 °C ± 0.36 °C (p < 0.001) in HM and SM, respectively, across the growing season. Effects of warming on microclimatic parameters are shown in Fig. 1. Warming increased soil temperature (ST) at 5 cm depth at different rates for the meadow types. HM being densely vegetated, as compared to SM, allowed lower solar radiations reaching ground inside OTCs and thus showed low and inconsistent soil warming across growing season, significant (p < 0.001) only during May, June and October with a mean increase of 1.0 ± 0.36 °C (p = 0.03). An increase in ST (2.19 °C ± 0.37 °C, p < 0.001) was consistent across the growing season in SM. Further, it was observed that soil warming efficiency lowered during the peak growing season at both meadows (Supplementary Figs. S1 and S2 online) probably due to decreased solar radiations reaching ground with increased plant growth. This effect was more profound at HM, having dense vegetation, leading to a significant decrease in ST (1.10 ± 0.39 °C, p = 0.006) under OTCs during August.

Consistent soil warming in SM decreased volumetric soil water content (SWC) by 38.9% (0.049 ± 0.01 m^3^ m^−3^ p =  < 0.001) across the growing season. (Fig. 1 and Table 1). SWC at HM showed no significant change under warming.

**Table 1:** Effects of experimental warming on various parameters in Herbaceous and Sedge Meadows across growing season.

Parameters	Herbaceous meadow		Sedge meadow	
	Control	Warming	Control	Warming
**Microclimate**				
AT (°C)	7.93 ± 0.26	9.42 ± 0.25***	7.59 ± 0.25	9.49 ± 0.25***
ST (°C)	11.13 ± 0.29	12.13 ± 0.21***	10.78 ± 0.27	12.97 ± 0.25***
SWC (m^3 ^m^−3^)	0.221 ± 0.008	0.203 ± 0.008^ ns^	0.175 ± 0.007	0.126 ± 0.007***
**Plant growth**				
AGB (g m^−2^)	352.65 ± 19.25	838.0 ± 96.06***	234.18 ± 25.6	463.69 ± 26.08***
Plant height (cm)	15.86 ± 2.42	29.0 ± 2.95**	9.36 ± 0.91	24.56 ± 1.70***
Leaf length (cm)	2.97 ± 0.25	4.28 ± 0.24***	2.34 ± 0.11	4.24 ± 0.16***
**Soil organic carbon**				
SOC (g Kg^−1^)	48.9 ± 3.89	46.14 ± 3.60^ ns^	37.80 ± 2.11	30.24 ± 1.53*
**Respiration**				
ER (µmol m^−2^ s^−1^)	4.47 ± 0.27	5.94 ± 0.34**	3.13 ± 0.17	4.01 ± 0.22**
SR (µmol m^−2^ s^−1^)	3.37 ± 0.21	3.51 ± 0.20^ ns^	2.44 ± 0.12	2.65 ± 0.14^ ns^
SR/ER ratio	0.80 ± 0.02	0.67 ± 0.03***	0.84 ± 0.02	0.72 ± 0.02**
**Respiration quotient (Q10)**				
Q10 of ER	4.1	4.5	3.3	3.6
Q10 of SR	3.7	4.0	3.2	4.0

Values are mean ± S.E.

Significant differences at p value * < 0.05, ** < 0.01 and *** < 0.001. ns reflects no significant difference.

*AT* air temperature; *ST* soil temperature; *SWC* volumetric soil water content; *AGB* above ground biomass; *SOC* soil organic carbon; *ER* ecosystem respiration; *SR* soil respiration.

### Effects of warming on plant growth and soil organic carbon

Experimental warming increased plant growth at both the meadows as evident from increased above-ground biomass (AGB), plant height and leaf length (Table 1). Increase in AGB under warming was 137.6% (485.5 ± 98 g m^−2^, p < 0.001) and 98% (229.5 ± 36.5 g m^−2^, p < 0.001) in HM and SM, respectively, during peak growing season. Plant height increased by 82.8% (13.14 ± 3.8 cm, p = 0.002) and 162.5% (15.2 ± 1.9 cm p < 0.001) whereas leaf length increased by 43.9% (1.3 ± 0.34 cm p < 0.0001) and 81.7% (1.9 ± 0.19 cm p < 0.001) under warming in HM and SM, respectively. Figure 1 shows warming effects on SOC across the growing season in HM and SM. Warming had no significant effect on SOC in HM. At SM, SOC decreased under warming significantly during May (17%, p = 0.023) and October (28%, p = 0.019) and non-significantly in June (17%, p = 0.105), July (14%, p = 0.315) and September (21%, p = 0.684) with mean decrease of 20% (p = 0.023) across growing season.

### Effects of warming on ER, SR and SR/ER ratio

**Figure 2:**
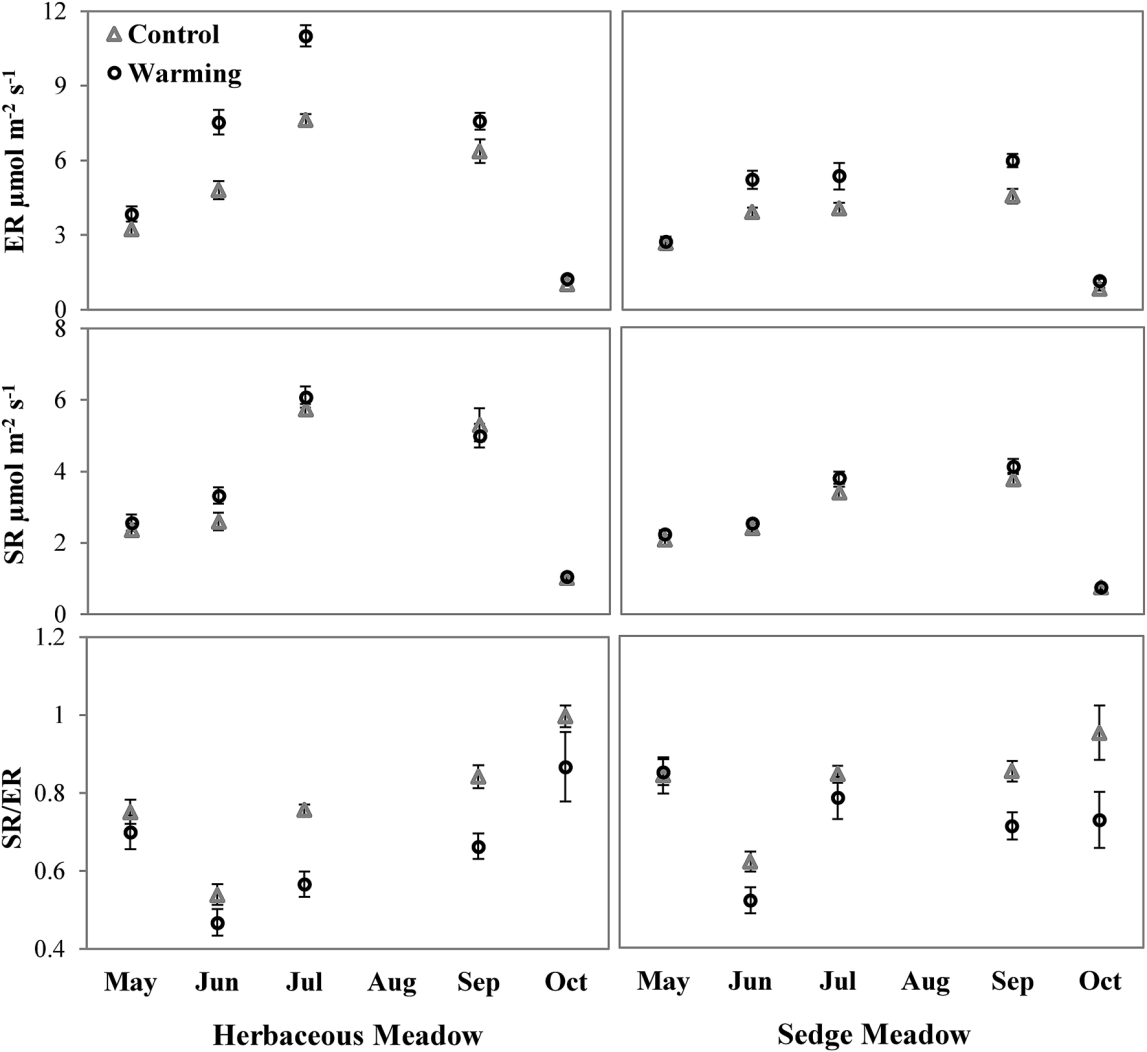
Warming effects on ecosystem respiration (ER) , soil respiration (SR) and SR/ER ratio at Herbaceous and Sedge Meadows. Data show mean ± S.E.

Mean ambient ER and SR were higher in HM by 42.9% (p = 0.001) and 37% (p = 0.007), respectively, across the growing season as compared to SM. Figure 2 shows the effects of experimental warming on ER, SR and SR/ER ratio. We observed an increase in ER by 33% (1.48 ± 0.34 µmol m^−2^ s^−1^, p = 0.002) and 28.2% (0.88 ± 0.28 µmol m^−2^ s^−1^, p = 0.003) under warming at HM and SM, respectively, across the growing season. Warming had no significant effect on SR across growing season at both the meadows except for an increase during June at HM by 27.7% (0.72 ± 0.37 µmol m^−2^ s^−1^, p = 0.043). SR/ER ratio decreased under warming at both meadows suggesting an increase in contribution from above-ground respiration across the growing season. Mean decrease was 16% and 14% at HM and SM, respectively.

### Factors affecting ecosystem and soil respiration

**Figure 3:**
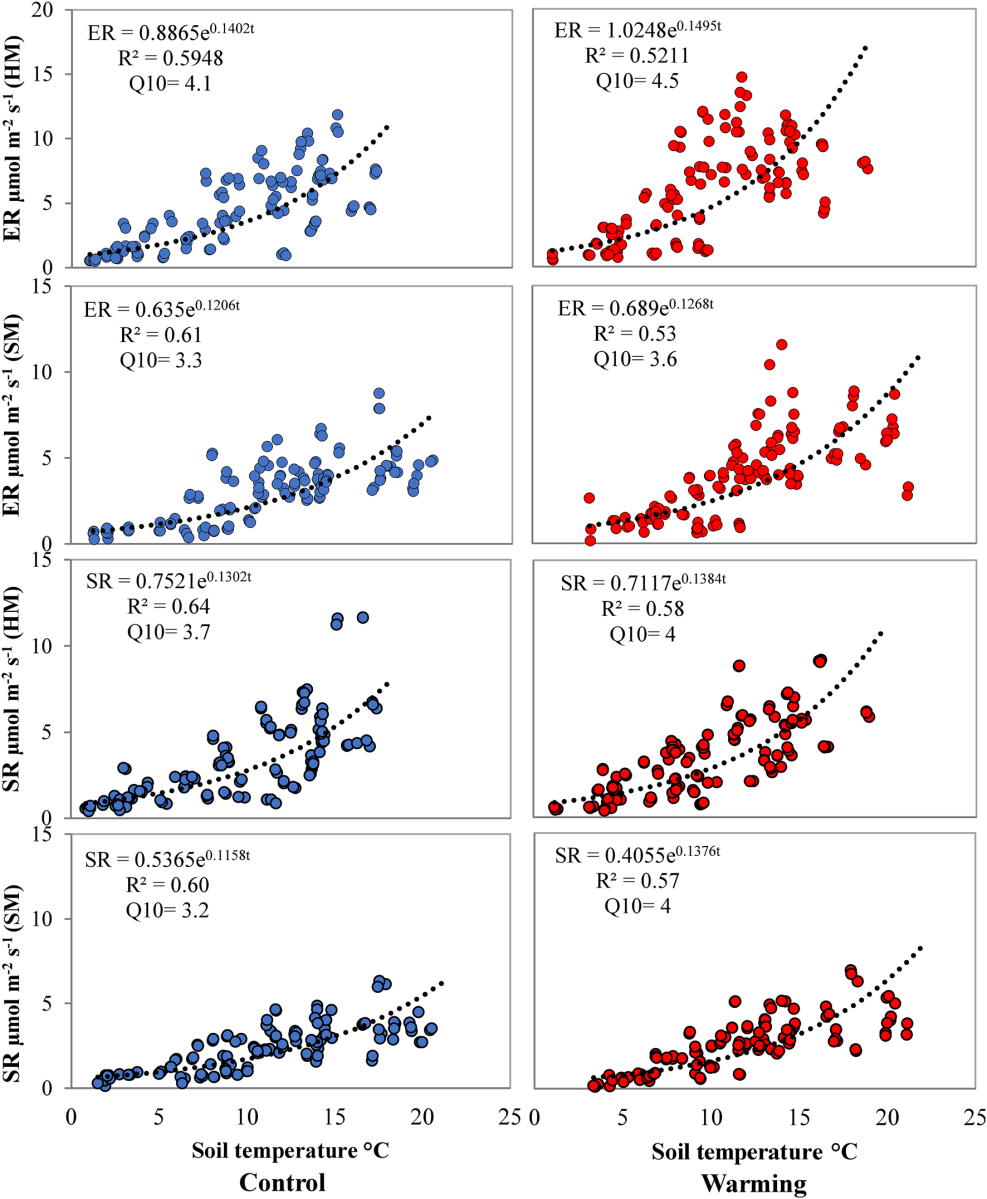
Exponential relationships between ecosystem respiration (ER) and soil temperature and soil respiration (SR) and soil temperature across growing season in control and warming plots in Herbaceous Meadow (HM) and Sedge Meadow (SM).

Both ER and SR increased exponentially (p < 0.001) with an increase in ST in both the meadows. Figure 3 shows the relationship of ER and SR with ST at 5 cm depth at both the meadows. ST explained variations in ER by 59% and 61% in control and 52% and 53% in OTCs at HM and SM, respectively. ST also explained variations in SR by 64% and 60% in control and 58% and 57% in OTCs at HM and SM, respectively. SWC had a weak relationship with both the respirations only in control explaining variations in ER by 6.9% (p = 0.004) and 6.6% (p = 0.005) and variations in SR by 4.1% (p = 0.027) and 8.3% (p = 0.001) in HM and SM, respectively. We did not observe effects of SOC on either ER or SR in any site. For detailed results of regression analysis, refer to Supplementary Table S1 online. Respiration quotient or Q10 of ER were 4.1 and 3.3, whereas Q10 of SR were 3.7 and 3.2 in HM and SM, respectively, in control. Experimental warming increased Q10 of ER and SR by 9.7% and 8.5%, respectively, in HM and 6.4% and 24.4%, respectively, in SM (Fig. 3 and Table 1).

## Discussion

Climate warming is expected to stimulate CO_2_ flux in alpine ecosystems, as elsewhere^39^. Our experimental study on warming effects on soil and ecosystem respiration (SR & ER) at two types of Himalayan alpine meadows does support this premise. Experimental warming of 1.49–1.9 °C (increase in AT inside OTCs) increased ER by 33% and 28% at HM and SM, respectively, (Table 1) across the growing season and is in agreement with several other studies in alpine region^44,45^. Interestingly, SR did not show a significant increase under warming on either type of meadow indicating that the increase in ER was an outcome of the warming-induced increase in above-ground respiration. This was supported by the fact that AGB increased by 137.6% and 98% and SR/ER ratio decreased by 16% and 14% at HM and SM, respectively, under warming at both the sites (Table 1). Increase in AGB under warming in our study is consistent but higher than the other studies from alpine meadows^12,29,45^. Warming advances growing phase by early snowmelt, shifts plant phenophases and expands growing season leading to higher plant productivity^46^. We observed both early onset and expansion of the growing season under warming (109th Julian day, 167–180 days) in comparison to control (145th Julian day, 128 days) in both the meadows (Supplementary Table S2 online). High plant productivity under warming elevates root respiration either directly by increased C allocation, assimilated by photosynthesis, to belowground or indirectly by increased root biomass^47–49^. We assume the same in our study however, the neutral response of SR (sum of root and heterotrophic respiration) to warming indicates that the increase in root respiration was offset by heterotrophic respiration.

Heterotrophic respiration originates mainly from microbial decomposition of labile soil carbon and root exudates and is limited by low temperature, substrate availability, soil moisture and alterations of microbial communities^50–53^. Initial warming, under adequate moisture and substrate availability, activates microbes resulting in a phase of accelerated respiration indicated by higher soil respiration rates and may cause C loss^54,55^. This stage is usually temporary as microbial communities tend to acclimatize to sustained warming by physiological adjustments or shifts in community composition due to (i) substrate depletion caused by accelerated respiration and (ii) warming induced moisture reduction, negatively impacting microbial processes and eventually decelerating respiration^51,56–58^. We observed that the OTCs that were recently installed in 2018 showed an increasing trend in SR under warming by 15% and 13% in HM and SM, respectively (Supplementary Table S3 online). This increase in SR may be attributed to an increase in heterotrophic respiration induced by initial warming. At SM, higher heterotrophic respiration under initial warming would have surpassed low carbon inputs from sedge plants eventually causing loss of SOC (as shown by 17% lower SOC during our first sampling in May 2019). However, substrate depletion and warming induced decrease in SWC (Table 1) might have restricted further microbial activity and attenuated warming-induced soil CO_2_ release which is evident in our sampling period. At HM, we did not observe any significant change in SOC under warming probably due to higher C allocation from herbaceous plants to below ground, in comparison to sedge plants, balancing any C loss from higher heterotrophic respiration under initial warming phase. However, herbaceous plants with increased height and leaf size acted as barrier to solar radiations reaching ground eventually causing low and inconsistent soil warming within OTCs (~ 1.0 °C). Inadequate soil warming might have limited microbial enzymatic activity and reduced heterotrophic respiration leading to a neutral response of SR to warming at HM^59^. The attenuation of SR and ER, at both the meadows, is also indicated in our analysis (Supplementary Table S3 online) showing higher respiration in initial warming (OTCs installed in May and October 2018) as compared to respiration under longer warming (OTCs installed in October 2016). Moreover, higher soil warming in HM (~ 2 °C) similar to that of SM is likely to increase heterotrophic respiration and eventually increase SR, given adequate SWC and SOC. This was evident during June when soil warming by ~ 2 °C at HM (Supplementary Fig. S2 online) increased SR by 27.7% (Fig. 2).

Consistent with various studies we observed an exponential relationship between ST and respiration rates (Fig. 3)^12,45,60^. This implies that respiration may increase with an increase in ST however, SR did not respond to warming in either site. This observation indicates that the effect of warming on SR is beyond a simple temperature response which we attribute to differential warming impacts on components of SR (autotrophic and heterotrophic respiration)^27,61^. Soil moisture (SWC) is also known to control ER and SR^12,62^. We observed a significant but weak relationship of SWC with ER and SR in control, indicating that SWC to some extent may regulate the magnitude of respiration response to warming in this region.

Respiration quotient (Q10) is an important parameter used to assess the temperature sensitivity of both ER and SR^63,64^. In our study Q10 of ER was 4.06 and 3.34 in HM and SM, respectively (Table 1). These values were well within the Q10 range (1.3–4.6) as reported by previous studies in alpine region^12,45^. However, Q10 of SR (3.2–3.7) was higher in comparison to the global average value (1.3–3.3)^65–67^. We hypothesized that warming would decrease Q10 of both ER and SR based on results from similar studies in other alpine regions^68–70^. But contrary to this, we observed an increase in Q10 of ER by 9.7% and 6.4% and Q10 of SR by 8.5% and 24.4% in HM and SM, respectively (Table 1). Increase in temperature sensitivity suggests that plant-derived respiration is the principal source of ER^45,69^.

## Conclusion

Our study provides an insight into the response of SR and ER to experimental warming (1.5–2 °C) across two major types of alpine vegetation in the Western Himalaya. Results suggest an increase in ecosystem C emissions via increased above-ground respiration under climate warming in the alpine meadows. Moreover, warming-insensitive SR is probably due to differential warming responses of its components and needs focus in future research. Increase in plant growth, as seen by increase in above ground biomass, leaf size and plant height suggests higher C sequestration and net C uptake under warming in these meadows.

## Methodology

### Study site

**Figure 4:**
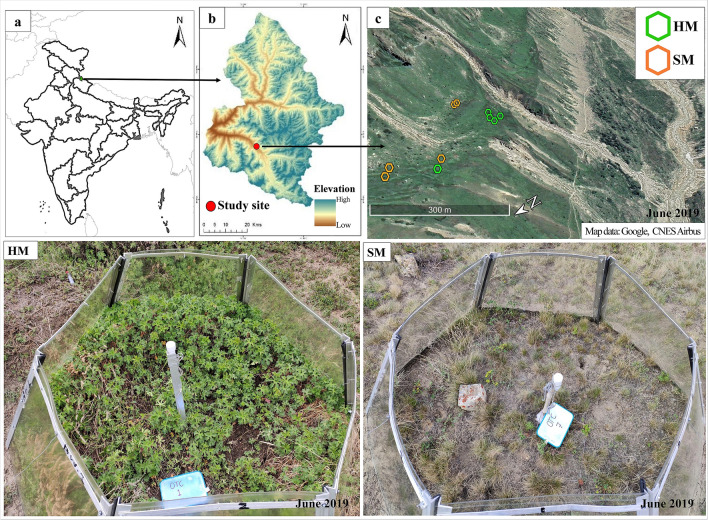
(**a**) State map of India, (**b**) Gangotri National Park and (**c**) Study site with open-top chambers at Herbaceous Meadow (HM) and Sedge Meadow (SM). Maps in figures a and b were generated with ArcGIS version 10.7 (ESRI, CA, USA, https://desktop.arcgis.com/en/arcmap/) and image in figure c was acquired using Google Earth Pro version 7.3.3.

The study was conducted in the alpine region of Gangotri National Park located in Western Himalaya, India (30°57′01.93″ N, 79°03′28.24″ E, 4000 m a.s.l) (Fig. 4). Mean annual precipitation is 1500 mm that occurs in form of rainfall during July to September and snowfall from December to May^70^. Mean annual temperature measured by data loggers at the study sites from November 2016 to October 2019 was 2.92 ± 0.36 °C. Vegetation of the study site can be categorized in two physiognomic types, viz., herbaceous meadow (HM) dominated by dicotyledonous herbs (*Geranium himalayense*, *Nepeta discolor, Artemisia gmelinii, Thalictrum alpinum, Cynoglossum wallichii* and *Galium rotundifolium*) and sedge meadow (SM) dominated by sedges (*Kobresia nepalensis* and *Carex* spp*.)* and few grasses eg. *Calamagrostis emodensis*. *G. himalayense* was also found in SM however in smaller number*.* Other associates common to both the types include *Polygonatum graminifolium*, *Persicaria polystachya*, *Euphorbia stracheyi* and *Astragalus candolleanus.* HM was characterized by dense vegetation and higher soil organic carbon and soil water content in comparison to SM (Table 1).

### Experimental design

In October 2016 homogeneous plots (2 m × 2 m) with similar plant communities and soil properties were selected in both meadow types (HM and SM) and assigned to treatments (control and warming). For warming treatment, a modified design of hexagonal open-top chamber (OTC) from that of Molau and Alatalo was used^71,72^. The chambers were constructed of 3 mm polycarbonate sheet with upper and bottom minimal diameter and height as 110 cm, 170 cm and 70 cm, respectively. Five OTCs were installed at each meadow (HM and SM) with a minimum distance of 10 m from each other^47^. A control plot with similar vegetation was marked adjacent to OTC and was fenced to avoid grazing. At HM three OTCs in October 2016 and two OTCs in October 2018 whereas at SM one OTC in October 2016 and four OTCs in May 2018, were installed as per feasibility. Our analysis shows similar warming effect on respiration by OTCs installed at different time in the respective meadow type (Supplementary Table S3 online). OTCs were left on plots all year around since installation and sampling was performed during the growing season in 2019 (from May to October).

### Microclimatic parameters

The height of the alpine vegetation at both the sites during peak growing period was below 40 cm. Therefore, the climatic variables influencing alpine plants (close to the ground) around experimental sites are referred as micro-climatic parameters here. Air temperature (AT) at 30 cm above ground and soil temperature (ST) at 5 cm depth was recorded by HOBO U23 Pro v2 data loggers (Onset Computer Corporation, Pocasset, MA, USA) at hourly interval. These data loggers were installed in each OTC (warming treatment) and one in each meadow site (control). In addition, ST and volumetric soil water content (SWC) at 5 cm depth were measured using hand-held soil temperature probe (6000-09TC, LICOR Inc., Lincoln, NE, USA) and GS1 soil moisture sensor (Decagon Devices, Inc., Pullman, WA) during respiration measurements.

### Above ground plant biomass measurement

Above-ground biomass (AGB) was measured once during peak growing season in July. Plants were clipped from soil collars inserted in both OTC and control for measuring soil respiration (covering a soil surface of 314 cm^2^) and collected in paper envelopes. Fresh weight of clipped plants was taken in-situ using a portable weighing balance (DW500 Nano, Danwer Scales Pvt. Ltd, India). The samples were then transported to the laboratory and dried at 65 °C for 72 h. Thereafter, dry weight was taken to assess above-ground plant biomass. To increase our understanding of how plant growth is affected by warming, we measured plant height and leaf length of *G. himalayense*, a common species in both meadow sites. For this, we took measurements from 5 random plants in each treatment plot.

### Soil sampling and soil organic carbon determination

Soil samples were collected at 5 cm depth using soil auger (diameter 5 cm) in sterilized plastic zip lock bags from 6 random places in each treatment and were pooled together (weighing ~ 5 g). The soil was homogenized, air-dried, sieved through 1 mm sieve and stored under 4 °C. Soil organic carbon (SOC) was estimated in duplicates using the potassium dichromate (K_2_Cr_2_O_7_) oxidation method^73^.

### Ecosystem and soil respiration measurement

Ecosystem respiration (ER) and soil respiration (SR) were measured using the LI-8100A Automated Soil CO_2_ Flux System (LICOR Inc., Lincoln, NE, USA). Opaque collars made of polyvinyl chloride (diameter = 20 cm and length = 11 cm) were inserted 2–3 cm in soil in each treatment. For SR, plants inside the collars were clipped from above 1 mm ground without disturbing the soil. Plots were left undisturbed for 24 h to let the ecosystem stabilize^45^. LI-8100–103 20 cm opaque survey chamber (diameter = 20 cm, centre height ~ 15 cm) was mounted on top of the soil collars to take respiration readings. The chamber height + collar offset (~ 25 cm) was adequate to cover most of the plants during ER measurement. However, during peak growing season plant height in some plots reached above 20 cm for which we gently folded the plants inside collars for the measurement duration. Respiration readings were taken in duplicates during May to July and in triplicates during September to October with an observation time of 120 s and dead band (initial stabilization time for which the readings are not incorporated into final efflux) of 15 s. Measurements were taken during May to October 2019 (except in August due to heavy rainfall) in each plot from 08:00 to 14:00 h at 3-h time interval which took 2–3 days to complete the whole cycle. Dates of measurements were 14–15th May, 20–22th June, 14–16th July, 5–7th September, and 24–26th October. Due to the remoteness of the study site and camping restrictions inside the National Park, measurements were taken once each month.

### Statistical analysis

Normal distribution and homogeneity of variance of the data were determined through Shapiro–Wilk and Levene's test, respectively. Since the data did not meet the assumption of normal distribution even after transformation, non-parametric Mann–Whitney U test was used to analyze the effects of warming on AT, ST, SWC, AGB, SOC, ER, SR and SR/ER ratio. We calculated SR/ER ratios to understand the shift in the contribution of SR to overall CO_2_ efflux under warming. All means and differences were reported in terms of monthly measurements (for ease of understanding) in the form of mean ± standard error and mean differences ± standard error, respectively. The relation between respiration (ER & SR) and ST was tested by fitting an exponential function, and Q10 values were calculated as given by Zhou et al*.*^60^^.^ Simple linear regression was conducted to determine the probable effect of SWC and SOC on respiration rates. Statistical analyses were performed in SPSS software (version 23.0, IBM, Chicago, IL, USA) and significant differences were assessed at the level p < 0.05.

## Supplementary Information

Supplementary Information 1.

Supplementary Information 2.
