# Supplementary material for: Experimental warming increases ecosystem respiration by increasing above-ground respiration in alpine meadows of Western Himalaya
Source: Sci Rep. 2021 Jan 29;11:2640. doi: 10.1038/s41598-021-82065-y (PMC7846769; doi:10.1038/s41598-021-82065-y)
Supplement: Supplementary file 2 — Supplementary Information 2. [file 41598_2021_82065_MOESM2_ESM.docx]

**Experimental warming increases ecosystem respiration by increasing above-ground respiration in alpine meadows of Western Himalaya**

**Authors**: Pankaj Tiwari, Pamela Bhattacharya, Gopal Singh Rawat, Ishwari Datt Rai, Gautam Talukdar^*^

**Table S1.** Regression models for the relationship of ecosystem respiration and soil respiration with soil temperature (ST), volumetric soil water content (SWC) and soil organic carbon (SOC). a and b are constants and f is the factor. Significant values (in bold) are determined at p<0.05.

|  | | | | **Herbaceous Meadow** | | | | **Sedge Meadow** | | | |
| --- | --- | --- | --- | --- | --- | --- | --- | --- | --- | --- | --- |
|  | **Factor** | **Plot** | **Equation** | **a** | **b** | **r^2^** | **p** | **a** | **b** | **r^2^** | **p** |
| **Ecosystem Respiration (ER)** | ST | control | ER=ae^bf^ | 0.887 | 0.140 | 0.595 | **<0.001** | 0.635 | 0.121 | 0.609 | **<0.001** |
|  |  | warming | ER=ae^bf^ | 1.025 | 0.150 | 0.521 | **<0.001** | 0.689 | 0.127 | 0.530 | **<0.001** |
|  | SWC | control | ER=bf+a | 3.073 | 6.314 | 0.069 | **0.004** | 2.377 | 4.281 | 0.066 | **0.005** |
|  |  | warming | ER=bf+a | 4.946 | 4.990 | 0.024 | 0.093 | 4.255 | -1.986 | 0.008 | 0.317 |
|  | SOC | control | ER=bf+a | 4.892 | -0.006 | 0.003 | 0.787 | 3.386 | -0.005 | 0.002 | 0.851 |
|  |  | warming | ER=bf+a | 7.761 | -0.033 | 0.054 | 0.261 | 6.228 | -0.071 | 0.112 | 0.102 |
|  |  |  |  |  |  |  |  |  |  |  |  |
| **Soil Respiration (SR)** | ST | control | SR=ae^bf^ | 0.752 | 0.130 | 0.640 | **<0.001** | 0.602 | 0.116 | 0.602 | **<0.001** |
|  |  | warming | SR=ae^bf^ | 0.712 | 0.138 | 0.584 | **<0.001** | 0.406 | 0.138 | 0.574 | **<0.001** |
|  | SWC | control | SR=bf+a | 2.507 | 3.876 | 0.041 | **0.027** | 1.830 | 3.508 | 0.083 | **0.001** |
|  |  | warming | SR=bf+a | 3.056 | 2.184 | 0.013 | 0.208 | 2.566 | 0.649 | 0.002 | 0.594 |
|  | SOC | control | SR=bf+a | 2.643 | -0.004 | 0.003 | 0.812 | 3.145 | 0.005 | 0.005 | 0.746 |
|  |  | warming | SR=bf+a | 3.405 | -0.024 | 0.036 | 0.366 | 4.117 | -0.011 | 0.019 | 0.507 |

**Table S2.** Effect of experimental warming on onset of growing season (day of year), duration of growing season (total number of days) and cumulative growing degree days (sum of mean daily air temperature during growing season) in herbaceous meadow (HM) and sedge meadow (SM). Base temperature = 5 °C

|  | | **Control** | **Warming** |
| --- | --- | --- | --- |
| **Onset of growing season DOY** | **HM** | 145th | 109th |
|  | **SM** | 145th | 109th |
| **Growing phase duration in days** | **HM** | 128 | 167 |
|  | **SM** | 127 | 180 |
| **Cumulative GDD °C** | **HM** | 1243.17 | 1649.163 |
|  | **SM** | 1179.386 | 1724.645 |

**Table S3.** Effects of experimental warming duration on ecosystem and soil respiration in Herbaceous (HM) and Sedge Meadow (SM) across growing season. Values are mean ± S.E. Significant differences at p value *<0.05, **<0.01 and ***<0.001. MD and ns indicates mean difference and non-significant respectively.

|  |  | **Ecosystem Respiration** | | | **Soil Respiration** | | |
| --- | --- | --- | --- | --- | --- | --- | --- |
|  |  | **Control** | **Warming** | **% MD** | **Control** | **Warming** | **% MD** |
| **HM** | **Oct-16** | 5.10 ± 0.37 | 6.31 ± 0.47 | 23.73^*^ | 3.86 ± 0.31 | 3.79 ± 0.27 | -1.81 ^ns^ |
|  | **Oct-18** | 3.51 ± 0.38 | 5.39 ± 0.50 | 53.56^**^ | 2.62 ± 0.24 | 3.08 ± 0.28 | 14.94 ^ns^ |
|  |  |  |  |  |  |  |  |
| **SM** | **Oct-16** | 4.23 ± 0.49 | 4.11 ± 0.49 | -2.84 ^ns^ | 3.28 ± 0.36 | 3.17 ± 0.41 | -3.35 ^ns^ |
|  | **May-18** | 2.85 ± 0.16 | 3.98 ± 0.25 | 39.65^***^ | 2.24 ± 0.12 | 2.52 ± 0.14 | 12.50 ^ns^ |


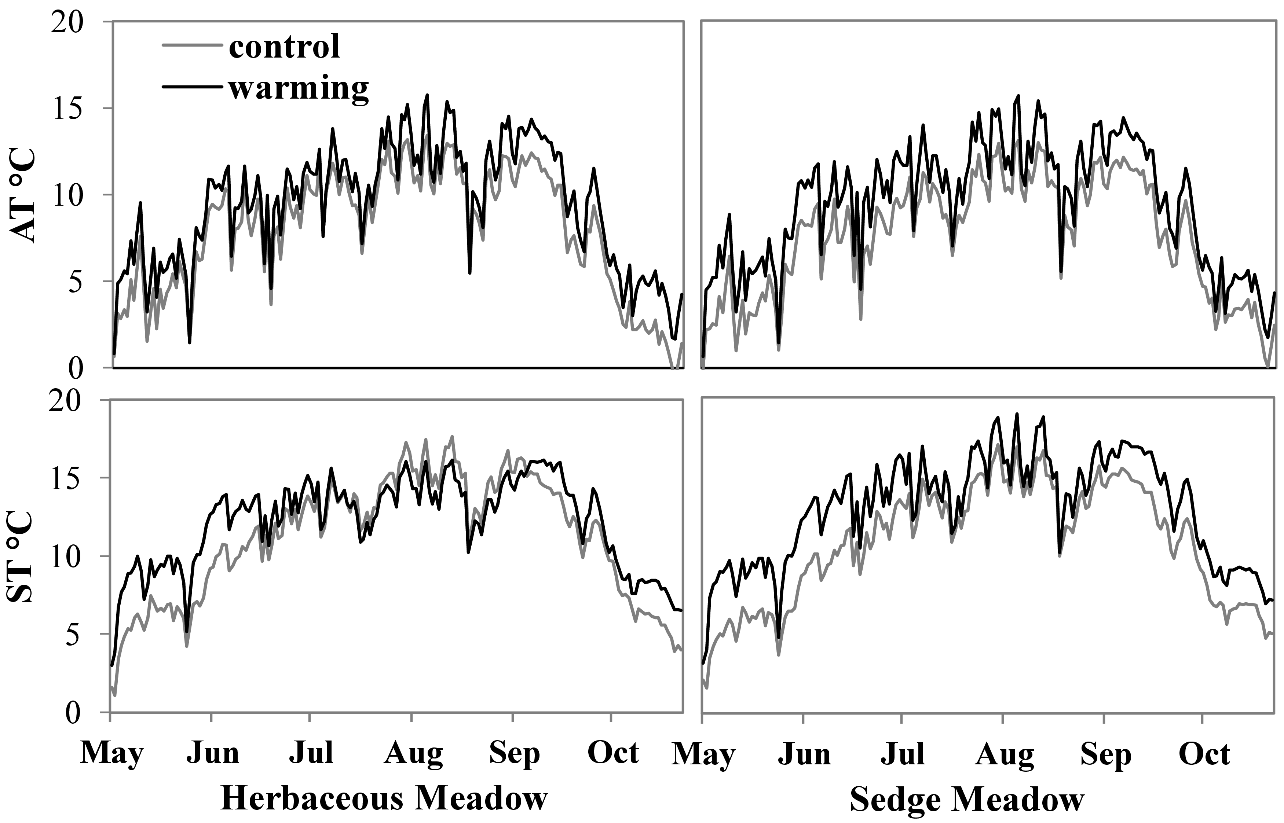


**Figure S1.** Variations in daily mean air temperature (AT) and soil temperature (ST) in Herbaceous and Sedge Meadow across growing season

**Figure S2.** Variations in air temperature (AT) and soil temperature (ST) under warming at herbaceous and Sedge Meadow across growing season. Positive and negative values show increase and decrease in temperature respectively under OTCs. Each data point represents mean ± S.E.
